# Identification of Twenty-Two New Complete Genome Sequences of Honeybee Viruses Detected in *Apis mellifera carnica* Worker Bees from Slovenia

**DOI:** 10.3390/insects15110832

**Published:** 2024-10-24

**Authors:** Laura Šimenc Kramar, Ivan Toplak

**Affiliations:** 1 Institute of Microbiology and Parasitology, Parasitology Unit, Veterinary Faculty, University of Ljubljana, Gerbičeva 60, 1115 Ljubljana, Slovenia; 2 Institute of Microbiology and Parasitology, Virology Unit, Veterinary Faculty, University of Ljubljana, Gerbičeva 60, 1115 Ljubljana, Slovenia; ivan.toplak@vf.uni-lj.si

**Keywords:** honeybee viruses, next-generation sequencing, complete genomes, phylogeny

## Abstract

In this study, honeybee viruses were identified in naturally infected honeybee colonies (*Apis mellifera carnica*). From nine selected samples of clinically affected and ten samples of healthy honeybee colonies, different strains of honeybee viruses were first detected using quantitative real-time RT-PCR methods. Twenty-two nucleotide sequences of the complete genomes of honeybee viruses were identified using the Illumina Next-Generation Sequencing (NGS) method: acute bee paralysis virus (ABPV) (n = 4), black queen cell virus (BQCV) (n = 3), chronic bee paralysis virus (CBPV) (n = 2), deformed wing virus (DWV) (n = 5), Lake Sinai virus (LSV) (n = 4), sacbrood bee virus (SBV) (n = 1), Apis rhabdovirus-1 (ARV-1) (n = 1), bee macula-like virus (BeeMLV) (n = 1) and Hubei partiti-like virus 34 (HPLV34) (n = 1). The nucleotide sequences of ABPV, BQCV, DWV and SBV are the first complete genomes of these viruses identified in Slovenia and they represent an important contribution to our understanding of the genetic diversity of honeybee viruses. ARV-1, BeeMLV and HPLV34 were detected and sequenced for the first time in Slovenia.

## Simple Summary

Twenty-two nucleotide sequences of the complete genomes of nine honeybee viruses were identified using the Illumina Next-Generation Sequencing method. The honeybee viruses were determined from clinically affected and healthy honeybee colonies. The nucleotide sequences of the acute bee paralysis virus, black queen cell virus, deformed wing virus and sacbrood bee virus are the first complete genomes of these virus species to be identified in Slovenia. Apis rhabdovirus 1, bee macula-like virus and Hubei partiti-like virus 34 were also detected for the first time in Slovenia. These new complete genome sequences represent an important contribution to our understanding of the genetic diversity of honeybee viruses.

## 1. Introduction

Honeybee viruses are among the most important pathogens of honeybees (*Apis mellifera)* which are frequently detected in beehives worldwide. Various honeybee viruses can also infect other wild pollinators such as bumblebees or even butterflies, the pathogenicity of which is not yet fully understood [1,2,3,4,5]. Honeybee viruses have largely been identified and described using various molecular methods, including Sanger sequencing and Next-Generation Sequencing (NGS) [6,7]. Honeybee virus infections can occur in both healthy and diseased colonies with mild or subclinical infection, although the combination of several factors, such as poor hive management, *Varroa destructor* infestation and the presence of bacterial infections, can contribute significantly to the rapid multiplication of honeybee viruses and thus to the loss of honeybees [8].

Pathogenic honeybee viruses are mainly isometric particles about 30 nm in size containing a single-stranded positive RNA molecule [9]. The sacbrood bee virus (SBV) and deformed wing virus (DWV) are classified in the genus *Iflavirus* (family *Iflaviridae*), and the acute bee paralysis virus (ABPV) and black queen cell virus (BQCV) are classified as *Cripavirus* (family *Dicistroviridae*), while the chronic bee paralysis virus (CBPV) has not yet been classified [10]. The Lake Sinai virus (LSV) is taxonomically assigned to the genus *Sinaivirus* and is genetically related to the chronic bee paralysis virus (CBPV) and viruses from the *Nodaviridae* family [11]. These viruses are the most frequently detected viruses in honeybee colonies in Europe and can infect larvae, pupae and adult bees. The exception is CBPV, which only infects adult bees [12,13].

The Carniolan bee (*Apis mellifera carnica*) is a subspecies of the western honeybee that has naturalized and adapted in the geographical area of Slovenia, in the southern part of the Austrian Alps and in the northern Balkan countries. The Carniolan bee is native to Slovenia and is a protected species by national law, so the introduction of other bee species is not permitted. The Carniolan bee is gentle and non-aggressive, has a good sense of direction and is less likely to drift into neighboring hives. In winter, it can survive with little honey stores, which is a good characteristic for areas with long winters. The increasing losses of honeybee colonies in our country over the last decade have led to great interest in honeybee pathology, and viruses have emerged as one of several candidates for these losses.

In one of the first studies in Slovenia, based on the detection of six honeybee viruses in clinically diseased Carniolan bees collected between 2007 and 2009, 40% of samples were found to be positive for ABPV, 83.3% for BQCV, 18.3% for CBPV, 70% for DWV, 1.7% for Kashmir bee virus and 8.3% for SBV [14]. Until this study, usually Sanger sequencing of partial genomes and phylogenetic analysis were performed in Slovenia [4,5,14,15].

The first larger study to characterize the overall nucleotide diversity among the ABPV sequences was carried out on field strains of ABPV, collected between 2007 and 2010 from the entire territory of Slovenia. Phylogenetic trees were constructed from 357 nucleotides of open reading frame 2 (ORF2) and 408 nucleotides of open reading frame 1 (ORF1) with 54 and 29 samples, respectively, showing 91.2–92.5% and 96.7–97.2% nucleotide identity [15].

The first genetic characterization of DWVs in Carniolan honeybees showed that they are genetically identical to those detected in DWV-infected *Varroa* mites. The phylogenetic analysis of 47 DWV-positive samples from honeybee colonies and 14 DWV-positive samples from *Varroa* mites collected throughout Slovenia between 2007 and 2013 confirmed the fact that *Varroa* is an important vector for transmission of the viruses between colonies [16].

Phylogenetic comparison of the partial RNA-dependent RNA polymerase gene region (557 nucleotides) revealed that the 26 Slovenian LSV strains are clustered into 3 genetic lineages: LSV lineage 1 (1 sample, 3.85%), LSV lineage 2 (10 samples, 38.46%) and LSV lineage 3 (15 samples, 57.69%) [17].

Also, 148 bumblebees collected throughout Slovenia in 2017 and 2018 and 180 honeybee samples were tested for 6 honeybee viruses. Direct sequencing of ABPV (n = 33), BQCV (n = 75), SBV (n = 25) and LSV (n = 25) revealed a nucleotide identity of 98.74 to 100% between the viruses detected in honeybees and bumblebees, confirming the evidence that honeybees and bumblebees are infected with genetically identical or closely related viral strains of the four endemic honeybee viruses in Slovenia [4].

Knowledge of the genome diversity of honeybee viruses is crucial to identify the pathogenicity and epidemiological routes of the viruses. Research in this field focuses on partial genome sequencing or whole-genome sequencing to determine the phylogeny associated with the disease pattern of infected honeybee colonies. In the last decade, the leading technology has often been NGS, which enables metagenomic screening to discover novel or unknown viruses in each sample of interest [6,17]. Several studies also led to the detection of new pathogens, confirming that NGS is an excellent tool for the detection of new honeybee viruses [6,7,17,18,19,20,21]. Prior to this study, only one complete CBPV and one complete LSV genome sequence from a collapsed honeybee colony in Slovenia had been identified and published [17,21].

The aim of this study is to identify the complete genome nucleotide sequences of different honeybee viruses from naturally infected honeybee colonies throughout Slovenia using the NGS method. Furthermore, a comparison with other complete genome sequences of honeybee viruses already available in GenBank is presented to display the differences and common features of honeybee viruses worldwide.

## 2. Materials and Methods

### 2.1. Sample Collection

Two hundred samples of worker honeybees (*Apis mellifera carnica*) were collected between the years 2018 and 2020 from different locations and covered the entire national territory of Slovenia. Each sample consisted of 10 bees of the same hive which were collected in sterile plastic bags and immediately stored at −70 °C until further examination.

**Figure 1:**
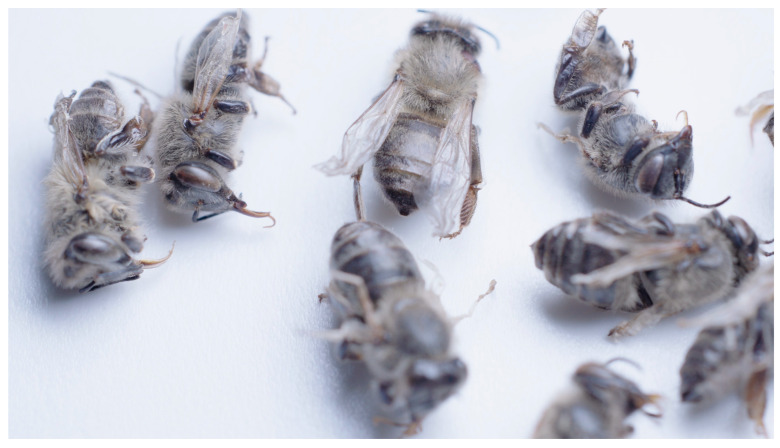
Dead worker honeybees (*Apis mellifera carnica*) with wing deformities, suspectedly caused by DWV. Author: Veterinary Faculty, University of Ljubljana.

The clinical status of each colony was determined prior to sampling. Clinically healthy bee colonies showed no clinical signs of viral infection or other signs of infectious diseases except for a slight *Varroa* infestation, which was detected prior to sampling. Clinically affected colonies were those colonies suspected of having a viral infection, such as those with dead bees in front of the hives, collapsing bees, weak colonies, trembling and/or paralysis of the bees, loss of color and hair and wing deformities (Figure 1).

### 2.2. Sample Preparation

Each sample consisted of 10 adult worker bees collected from 1 honeybee colony. Ten bees of each sample were placed in Ultra-Turrax DT-20 tubes (IKA, Königswinter, Germany) with 10 mL RPMI 1640 medium (Gibco, Paisley, UK) and incubated for 30 min at room temperature. The samples were homogenized and centrifuged at 2500× *g* for 15 min. A 2 mL amount of the supernatant from each sample was kept as a suspension for further viral RNA extraction.

### 2.3. RNA Extraction

Total RNA was extracted from a 200 µL suspension of each sample using the MagMax Core Kit with King Fisher Instrument (Thermo Fisher Scientific, Waltham, MA, USA) and diluted in 80 µL elution buffer of the same kit. The concentration of extracted RNA was measured using the QubitTM 3.0 fluorometer (Thermo Fisher Scientific, USA).

### 2.4. Quantitative RT-qPCR Assays

Initially, samples from 200 colonies (*Apis mellifera carnica*) were tested for the detection of 6 honeybee viruses by quantitative real-time RT-PCR (RT-qPCR) methods as previously described [22]. Briefly, primers, TaqMan probes and quantification standards for ABPV [23], BQCV [24], CBPV [13], DWV [25], LSV [22] and SBV [22] were used from previously published protocols. Reverse transcription with quantitative RT-qPCR assay was performed in a single step using the Quanti Nova Pathogen +IC kit (Qiagen, Hilden, Germany). The RT-qPCR mixture consisted of 5 µL Quanti Nova Master Mix, 2 µL 10× Internal Control (IC) Probe Assay, 1 µL IC (1:100), 4.5 µL deionized water, 1 µL forward primer (200 nM), 1 µL reverse primer (200 nM) and 0.5 µL probe (100 nM) as well as 5 µL extracted RNA with a 20 µL total final volume. Thermal cycling was performed on an Mx3005P thermal cycler (Stratagene, La Jolla, San Diego, CA, USA) under the following conditions: 20 min at 50 °C, 2 min at 95 °C, followed by 45 cycles of 15 s at 95 °C, 30 s at 60 °C and 30 s at 60 °C. In each run, the positive control was included, which was prepared as a mixed suspension of previously determined positive field samples of six different viruses (ABPV, BQCV, CBPV, DWV, LSV3 and SBV). Known copy numbers of the standard for each virus were prepared at 10-fold dilutions from 10^−3^ to 10^−7^ and added in each RT-qPCR run. The exact number of RNA virus molecules in each sample was calculated for positive samples according to cycle quantification (Cq) obtained via real-time RT-PCR methods and using the standard curve for each of the six honeybee viruses. The results for each sample were analyzed using MxPro-Mx3005P v4.10 software (Stratagene, La Jolla, USA) and the exact copy number was determined.

### 2.5. Selection of Samples for NGS

According to the results of testing 200 samples via 6 RT-qPCR methods, 19 samples were identified containing high viral loads (Cq < 20) of 1 or several honeybee viruses, and these samples were selected for further processing by NGS. The positive results from these 19 selected positive samples were confirmed also by conventional RT-PCR method, followed by Sanger sequencing for ABPV, BQCV, CBPV, DWV, LSV3 and SBV as previously described (Table S1) [4].

### 2.6. Library Preparation

Library preparation and sequencing were performed using Illumina technology at the commercial center Novogene (Cambridge, UK). Libraries were sequenced from both sides (2 × 150 base pairs) on the NovaSeq 6000 system (Illumina, San Diego, CA, USA) according to the appropriate protocol.

### 2.7. Quality Control and Genome Assembly

The sequenced reads were quality checked and trimmed using the FastQC v0.11.8. tool and Cutadapt version 1.18 [26]. Only the genomes with good coverage (>30) across the whole genome were included in the analysis, so all assemblies were of high quality. Viral genomes from our samples were reconstructed based on read mapping to the reference genome GenBank (NCBI, Montgomery, MD, USA). The list of reference genomes and identity with the reference genome are provided in Table S2. Reads were mapped to the reference genome using Geneious Alignment implemented in the Geneious Prime Version 2019.2.3 Software Suite (Biomatters Ltd., Auckland, New Zealand) with default parameters. The consensus sequences of the genomes were generated with MAFFT v.7.450 [27]. The program MEGA version 7.0.25 was used for phylogenetic analysis, and the trees were created using the maximum likelihood method with bootstrapping of 1000 replicates. The best model was chosen for each alignment using the “select best-fit substitution model” feature.

## 3. Results

Twenty-two complete genome sequences of honeybee viruses were identified and assembled using the NGS method from a total of ten samples from clinically healthy and nine samples from clinically affected honeybee colonies. Four complete genome sequences belonged to ABPV, three to BQCV, two to CBPV, five to DWV, four to LSV and one complete genome sequence each to SBV, ARV-1, BeeMLV and HPLV34. The complete genome sequences are available in the NCBI database under Bio Project: PRJNA889612 (Table 1). Single or multiple complete genome sequences were identified from the same tested samples. Samples with multiple complete genome sequences were all collected from clinically affected honeybee colonies (Table 1).

**Figure 2:**
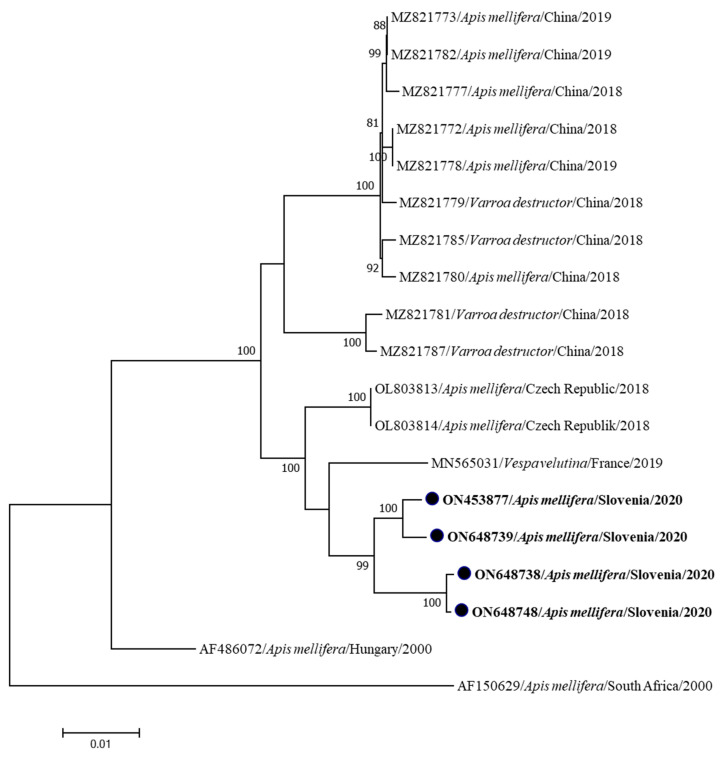
The phylogenetic comparison of 4 Slovenian nucleotide sequences of complete genomes of ABPV generated together with 15 complete genome sequences available in GenBank. Four identified ABPV sequences (ABPV 366/2020-ON453877, ABPV 377/2020-ON648739, ABPV 376/2020-ON648748 and ABPV 386/2020-ON648738) are marked with dots and bold text. The maximum likelihood tree was computed based on the TN93+G model with a bootstrap support of 1000 repetitions.

The four new complete genome sequences of ABPV were identified (ABPV 366/2020-ON453877, ABPV 377/2020-ON648739, ABPV 376/2020-ON648748 and ABPV 386/2020-ON648738), all from samples of clinically affected honeybees collected in Slovenia in 2020. The samples were collected from four different locations in Slovenia, separated by a minimum of 15 km and a maximum of 107 km (Mengeš, Semič, Žalec and Ljubljana). The comparison of the four identified Slovenian ABPV strains showed a nucleotide identity of 98.33 to 99.85%. Phylogenetic comparison of the determined sequences with genome sequences available in GenBank showed the closest relationship with ABPV genome sequences collected from *Apis mellifera* in the Czech Republic, OL803814 (nucleotide identity of 97.65 to 98.29%), and with ABPV genome sequences collected from *Vespa velutina* in France, MN565031 (nucleotide identity from 97.53 to 97.76%) (Figure 2).

**Figure 3:**
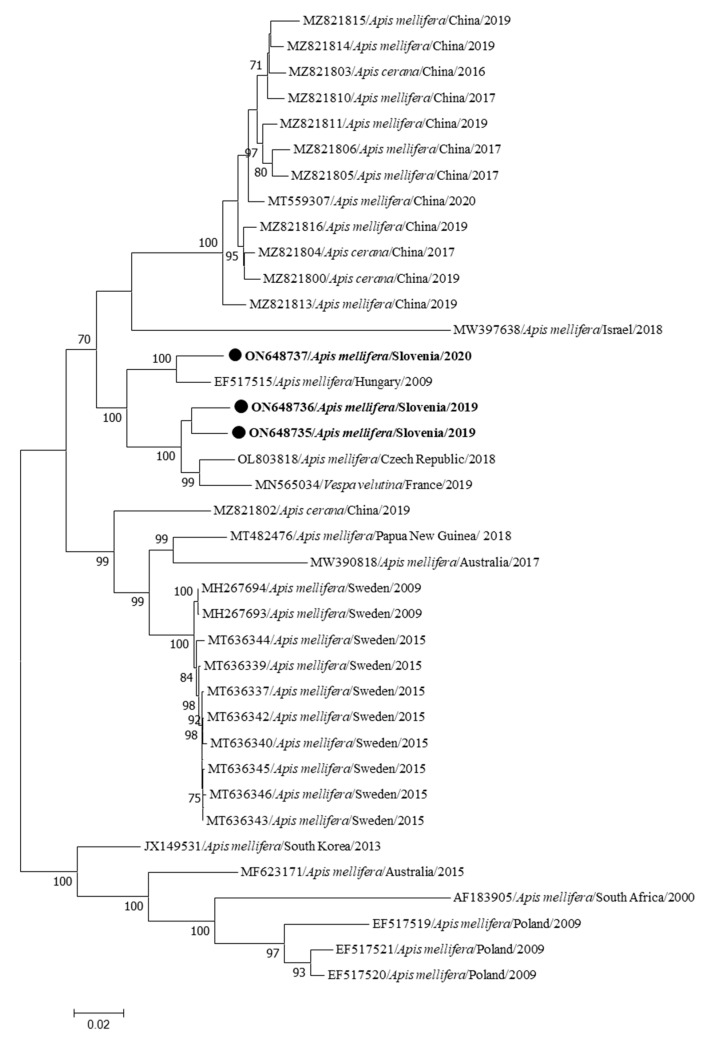
The phylogenetic comparison of 3 Slovenian nucleotide sequences of complete genomes of BQCV, together with 35 complete genome sequences available in GenBank. Three identified BQCV sequences (BQCV 377/2020-ON648737, BQCV LS90/2019-ON648736 and BQCV 336/2020-ON648735) are marked with dots and bold text. The maximum likelihood tree was computed based on the TN93+G model with a bootstrap support of 1000 repetitions.

The nucleotide sequences of the first three identified complete genomes of BQCV detected in *Apis mellifera carnica* in Slovenia were collected from clinically affected workers (BQCV 377/2020-ON648737, BQCV LS90/2019-ON648736 and BQCV 336/2020-ON648735) at three different locations in Slovenia (Semič, Radovljica and Mirna Peč, which are at least 37 km and at most 157 km apart). The three Slovenian BQCV sequences showed a nucleotide identity of 94.28 to 97.24% to each other. Comparison of the identified BQCV sequences with genome sequences available in GenBank showed that the most closely related (from 93.53 to 97.01%) are BQCV complete genome sequences from Hungary (EF517515) and the Czech Republic (OL803818), both detected in *Apis mellifera*, and the BQCV strain detected in *Vespa velutina* in France (MN565034) (Figure 3).

**Figure 4:**
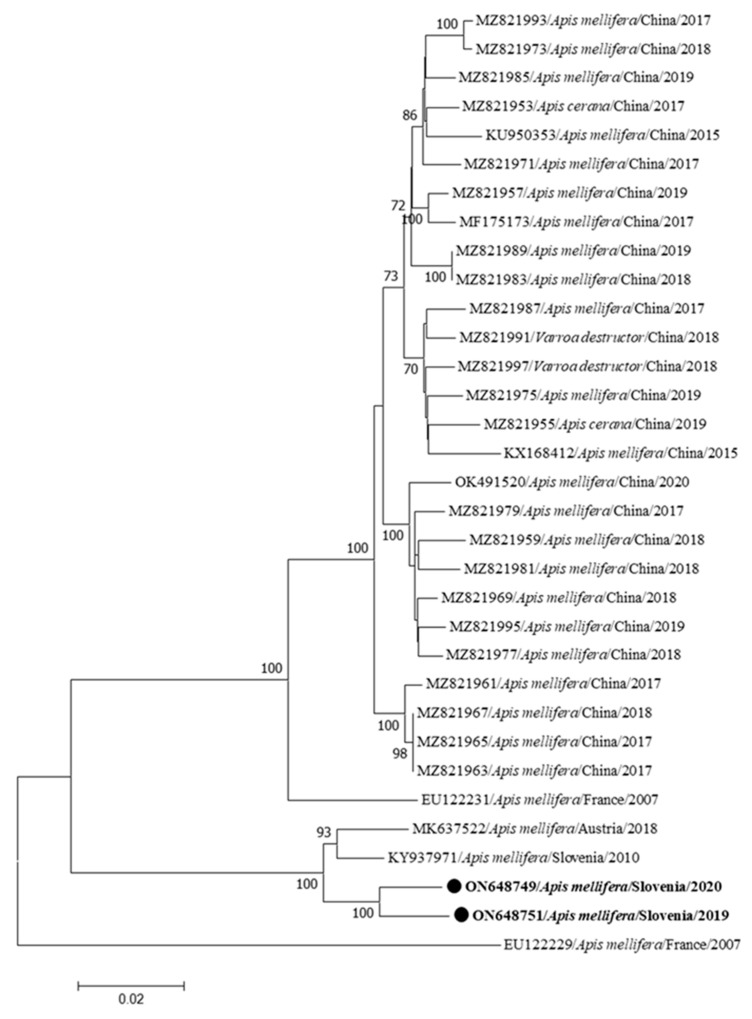
Phylogenetic comparison of the 33 nucleotide sequences RNA1 of the complete genomes of CBPV. The nucleotide sequences of two complete genomes of the RNA1 fragment (CBPV RNA1 376/2020-ON648749, CBPV RNA1 341/2019-ON648751) identified in this study are marked with dots and bold text. The maximum likelihood tree was computed based on the T93+G+I model with a bootstrap support of 1000 repetitions. RNA1 is the first fragment of CBPV genome.

The identified nucleotide sequences of two complete genomes of CBPV from Slovenia were collected from clinically affected *Apis melifera carnica* workers at two different locations in Slovenia (Žalec and Videm near Ptuj), which are 78 km apart. Two Slovenian CBPVs share 97.84% nucleotide identity in the RNA1 fragment (CBPV RNA1 376/2020-ON648749, CBPV RNA1 341/2019-ON648751). These two complete CBPV genome sequences are most closely related to the complete CBPV genome sequence detected in clinically affected *Apis mellifera carnica* in Slovenia in 2010 with 97.18 to 97.25% nucleotide identity in RNA1 (M92/2010-KY937971) and to the second most closely related strain of CBPV from Austria (AUT-17-MK637522) with 96.84 to 96.88% nucleotide identity in RNA1 (Figure 4).

**Figure 5:**
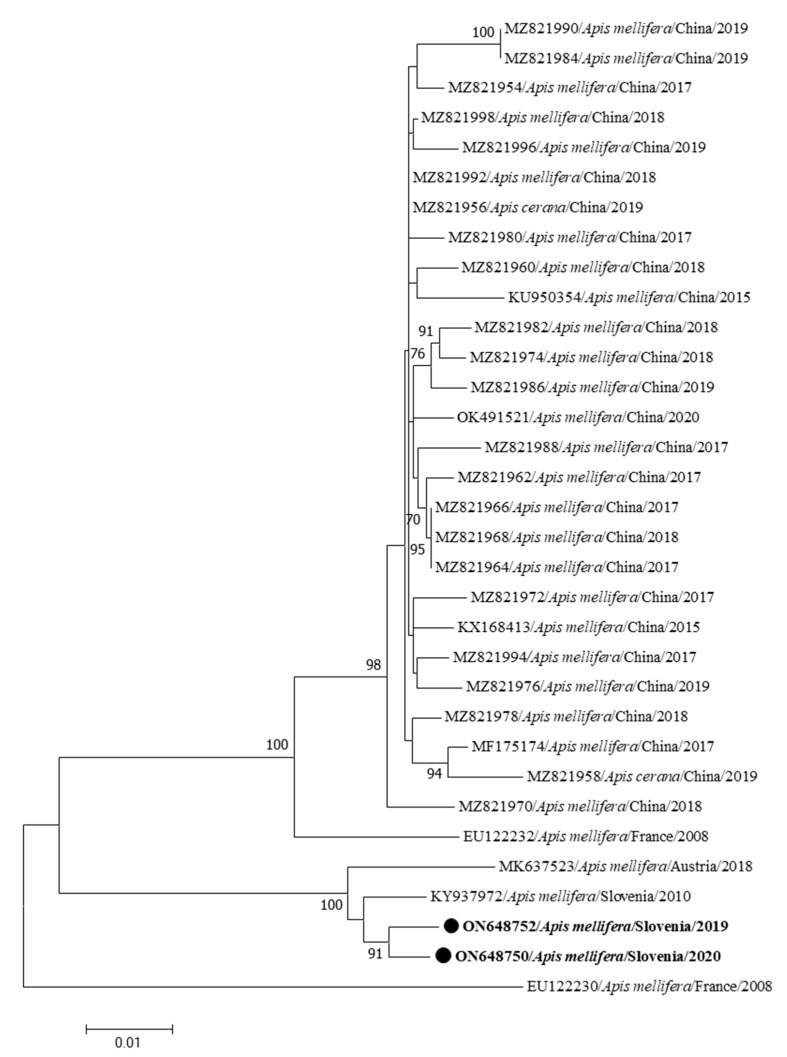
Phylogenetic comparison of the 33 nucleotide sequences RNA2 of the complete genomes of CBPV. The nucleotide sequences of two complete genomes of the RNA2 fragment (CBPV RNA2 341/2019-ON648752, CBPV RNA2 376/2020-ON648750) identified in this study are marked with dots and bold text. The maximum likelihood tree was computed based on the GTR+G+I model with a bootstrap support of 1000 repetitions. RNA2 is the second fragment of CBPV genome.

Two identified Slovenian CBPV sequences have 98.81% nucleotide identity in the RNA2 fragment (CBPV RNA2 341/2019-ON648752, CBPV RNA2 376/2020-ON648750). Most closely related to these two CBPV RNA2 genome sequences is the CBPV strain detected in 2010 in Slovenia in clinically affected *Apis mellifera carnica*, with 98.50 to 98,72% nucleotide identity in the RNA2 fragment (M92/2010-KY937972), while the second closest related strain is the CBPV strain from Austria (AUT-17-MK637523) with 97.36 to 97.62% nucleotide identity in RNA2 (Figure 5).

**Figure 6:**
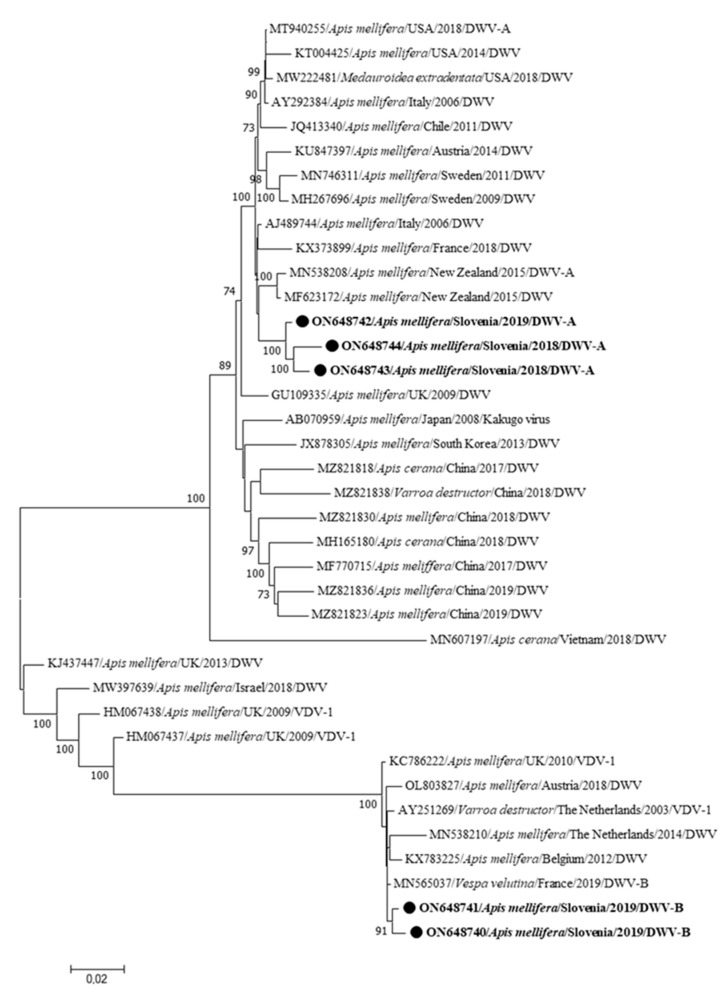
Phylogenetic comparison of 38 nucleotide sequences of complete genomes of DWV. The nucleotide sequences of three complete genomes of DWV-A (DWV 341-2/2019-ON648742, DWV LS13/2018-ON648743 and DWV LS24/2018-ON648744) and two complete genomes of DWV-B (DWV 336/2019-ON648740 and DWV 341-1/2019-ON648741) identified in this study are marked with dots and bold text. The maximum likelihood tree was computed based on the GTR+G+I model with a bootstrap support of 1000 repetitions.

The five complete genome sequences of DWV described in this study are the first complete genome sequences of DWV identified in Slovenia. Three sequences belong to DWV-A (DWV 341-2/2019-ON648742, DWV LS13/2018-ON648743 and DWV LS26/2018-ON648744) and two sequences to DWV-B (DWV 336/2019-ON648740 and DWV 341-1/2019-ON648741) (Figure 6). DWV-positive samples were collected at three different locations in Slovenia (Ljubljana, Videm near Ptuj and Mirna Peč), which are between 61 and 134 km apart. In a positive sample labeled DWV 341/2019, co-infection with two different DWV strains (DWV-A-341-2/2019-ON648742 and DWV-B-341-1/2019-ON648741, Figure 5) was detected. The three DWV-A sequences described in this study have a nucleotide identity of 98.37 to 98.89%. The two DWV-B sequences described in this study have a nucleotide identity of 99.30%. The difference between the Slovenian DWV-A and DWV-B genome sequences is between 14.52 and 15.48%. The genome sequence in GenBank most closely related to the DWV-A sequences identified in this study is the sequence AY292384 from Italy, which has a nucleotide identity of 97.26 to 97.87% with the DWV-A genome sequences from Slovenia. The genome sequence from GenBank that is most closely related to the DWV-B sequences is the sequence MN565037 from France, which has a nucleotide identity of 99.26 to 99.48% with the DWV-B sequences from Slovenia.

**Figure 7:**
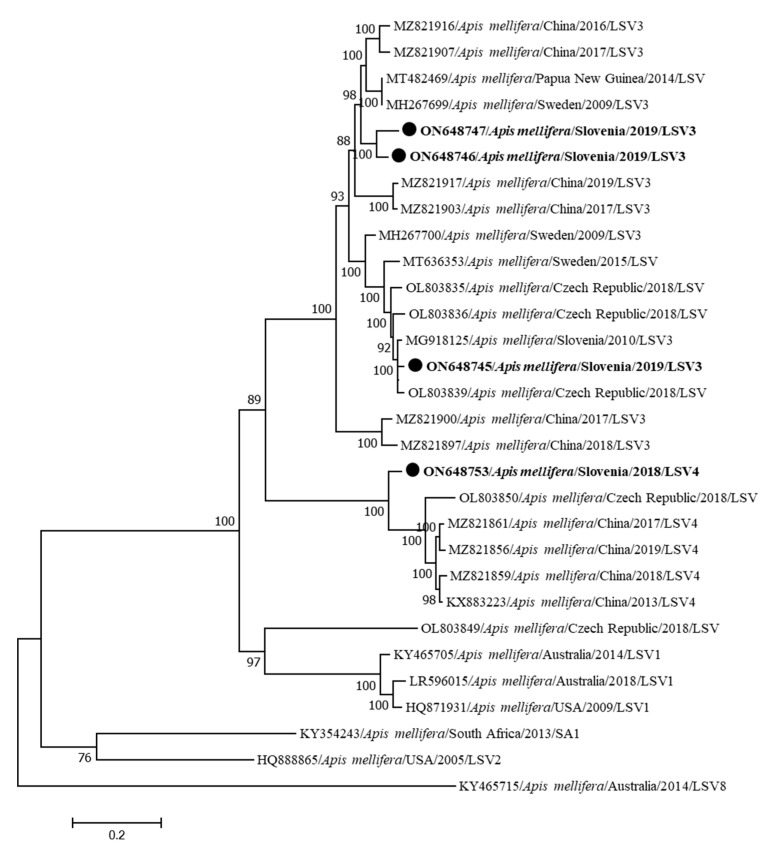
Phylogenetic comparison of 30 nucleotide sequences of complete genomes of LSV. The nucleotide sequences of three complete genomes of LSV3 (LSV3 LS48/2019-ON648745, LSV3 LS74/2019-ON648746 and LSV3 LS 81/2019-ON648747) and one complete genome of LSV4 (LSV4 LS21/2018-ON648753) obtained in this study are marked with dots and bold text. The maximum likelihood tree was computed based on the GTR+G+I model with a bootstrap support of 1000 repetitions.

Four complete genome sequences of the Slovenian LSV were identified from clinically healthy bees (LSV3 LS18/2019-ON648745, LSV3 LS74/2019-ON648746, LSV3 LS1812019-ON648747 and LSV4 LS21/2018-ON648753), showing between 76.41 and 93.58% nucleotide identity. Three genome sequences (ON648745, ON648746 and ON648747) are clustered together with LSV3 strains. ON648745 has 97.37% nucleotide identity with the previously determined complete genome from Slovenia M92/2010 (MG918125), while ON648746 and ON648747 have 93.65 nucleotide identity, and the most closely related strain in GenBank is MH267699 with 90.65% nucleotide identity, which was discovered in Sweden in 2009. A genome sequence from Slovenia (ON648753) belongs to the LSV4 cluster and is most closely related (88.35%) to the LSV strain detected in China (MZ821861) (Figure 7). This is the first identification and determination of the first complete genome of LSV4 in Slovenia.

**Figure 8:**
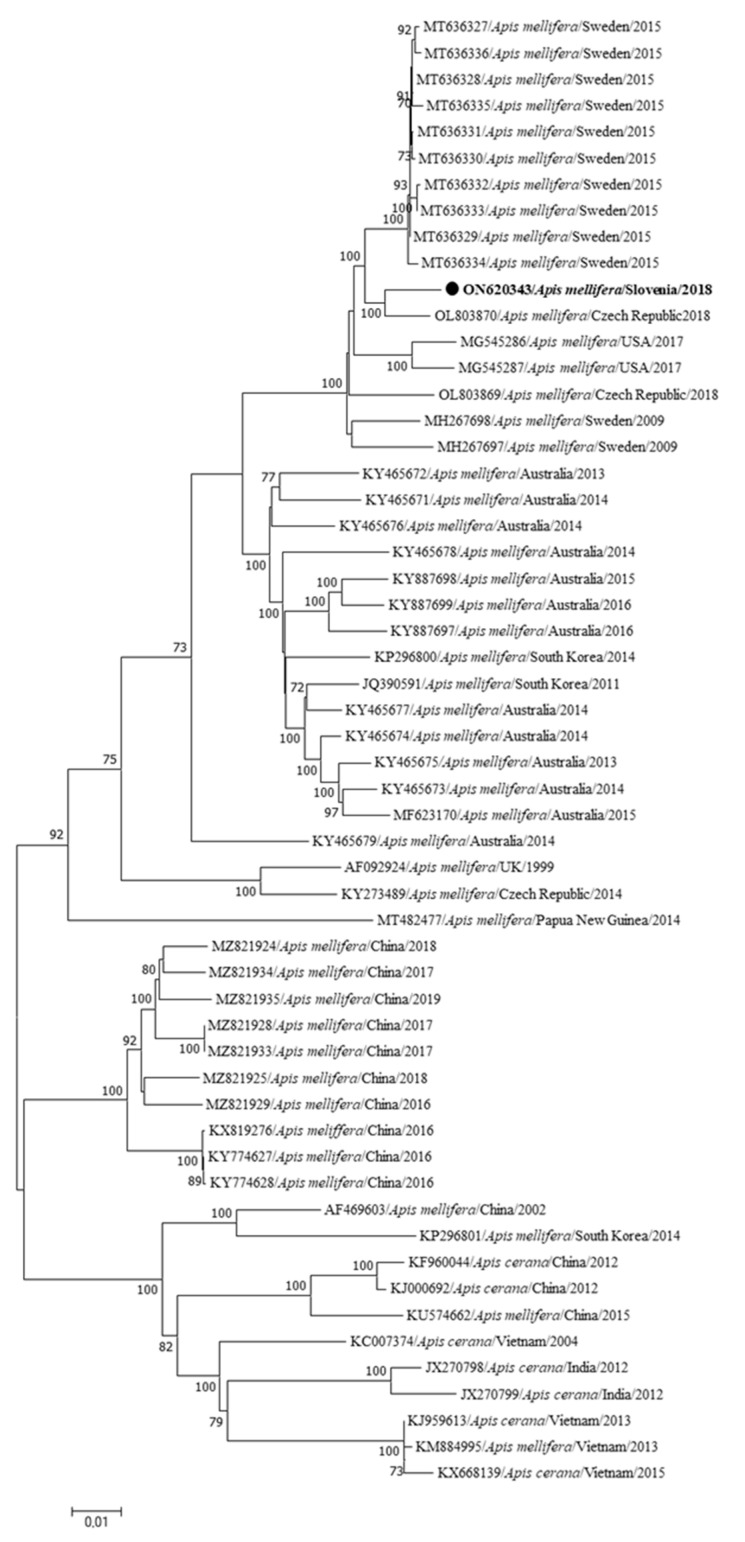
Phylogenetic comparison of the 56 nucleotide sequences of the complete genomes of SBV. The nucleotide sequence of the complete genome identified in this study (SBV LS20/2018-ON620343) is marked with a dot and bold text. The maximum likelihood tree was computed based on the GTR+G+I model with a bootstrap support of 1000 repetitions. The length of the branches is proportional to the number of substitutions per site.

The first complete genome sequence of SBV (LS20/2018-ON620343) was identified from a healthy honeybee colony from Murska Sobota, Slovenia, collected in November 2018. The complete genome sequence of SBV (LS20/2018-ON620343) is most closely related to the sequence SBV9C/2018-OL803870 from the Czech Republic (98.01% nucleotide identity) (Figure 8).

**Figure 9:**
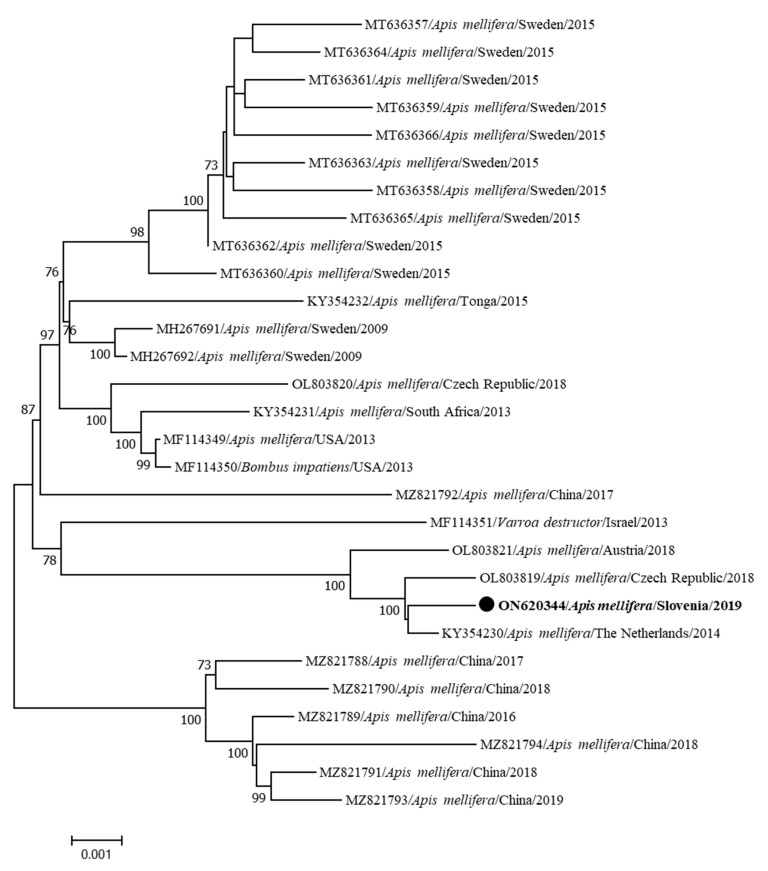
Phylogenetic comparison of 29 nucleotide sequences of the complete genomes of ARV-1. The nucleotide sequence of a complete genome identified in this study (ARV-1 341/20219-ON620344) is marked with a dot and bold text. The maximum likelihood tree was computed based on the TN93+G model with a bootstrap support of 1000 repetitions.

The first identified sequence of the complete genome of ARV-1 in Slovenia (ARV-1 341/2019-ON620344) was identified from an infested honeybee colony collected in Videm near Ptuj in February 2019 and is most closely related (99.78%) to the sequence of genome KY354230 collected in the Netherlands in 2014 (Figure 9). This is the first detection of ARV-1 in Slovenia.

**Figure 10:**
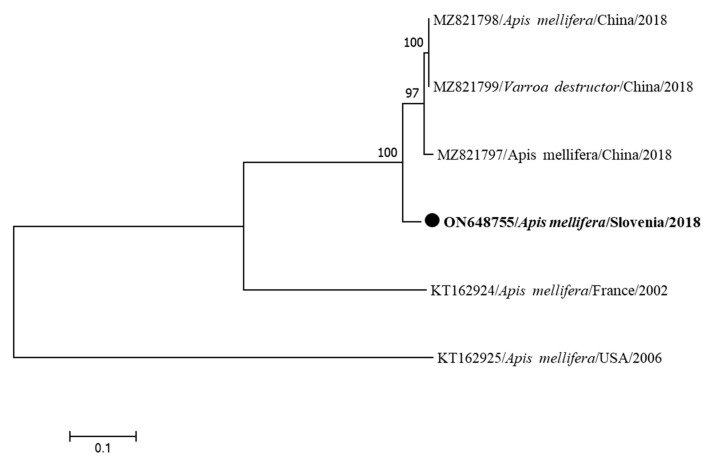
Phylogenetic comparison of 6 nucleotide sequences of the complete genomes of BeeMLV. The nucleotide sequence of a complete genome identified in this study (BeeMLV LS13/2018-ON648755) is marked with a dot and bold text. The maximum likelihood tree was computed based on the HKY+G model with a bootstrap support of 1000 repetitions.

The first identified nucleotide sequence of the complete genome of BeeMLV in Slovenia (BeeMLV LS13/2019-ON648755) was collected in 2019 from a clinically healthy bee colony in Ljubljana and is most closely related (93.63%) to the sequence from China (MZ821799) collected in 2018 (Figure 10). This is the first report of BeeMLV in Slovenia.

The first identified nucleotide sequence of the complete genome of HPLV34 in Slovenia (HPLV34 377-ON486754) was collected in 2020 from a clinically diseased honeybee colony in Semič and is most closely related (99.18%) to the sequence from China (OR496448) collected in 2023 from Apis mellifera This is the first report of HPLV34 in Slovenia.

## 4. Discussion

Slovenia is an important producer of honey and honeybee products. Queen bees of the Slovenian indigenous honeybee breed *Apis melliffera carnica* are distributed all over the world and are known for their peaceful character and high honey production potential [20]. The leading method of the last decade for analyzing complete genomes is the Illumina NGS method, which was also used in this study. Because only two complete genomes of honeybee viruses have been determined in Slovenia so far [17,21] and a still low number of each virus species was available in GenBank, the new identified complete genomes from Slovenia extend the diversity of honeybee viruses worldwide. The complete genome sequences of honeybee viruses ABPV, ARV-1, BeeMLV, BQCV, DWV, HPLV34 and SBV were identified in this study for the first time in Slovenia, which is an important contribution to the scientific community.

The previously observed differences in expected viral loads [22] might influence the NGS sensitivity (successful identification of complete genomes). The results of this study showed that at least one but also two or even several different complete genomes of honeybee viruses can be detected in the same sample when selecting samples with high viral load for NGS (Table 1). NGS is also excellent technology for the detection of genetically different strains of the same type of virus or previously not identified viruses present in the same colony, compared to other methodologies (RT-PCR, quantitative RT-PCR).

All four new complete genome sequences of ABPV from Slovenia were identified from the samples of clinically affected bee colonies. Phylogenetic analysis showed that these genome sequences are closely related, but, according to a previous observation, determined by partial genome sequencing, an even higher diversity of Slovenian ABPV field strains might be expected when the number of samples tested by NGS is increased [15]. This is suggesting that several additional ABPV strains are circulating in our territory and remain to be identified in complete genomes. Most closely related to the identified four Slovenian ABPV strains are strains from the Czech Republic (OL803813, OL803814) and France (MN565031), confirming that genetically similar ABPV sequences might be described also in our neighboring countries (Austria, Italy, Hungary, Croatia) in the future.

The identified complete genome sequences of BQCV expand the known diversity of BQCV field strains for European countries and the data based on a partial sequencing study in Slovenia [16]. Three complete genome sequences of BQCV showing between 94.28 and 97.24% nucleotide identity, similar to what was observed in the comparison of the diversity based on the partial genome BQCV sequence, were detected between honeybee- and bumblebee-positive samples in Slovenia [4]. The most closely related genome sequences from GenBank were from the Czech Republic (OL803818), Hungary (EF517515) and France (MN565034), which is expected due to historical connections and geographical proximity.

The CBPV genomes are special compared to other RNA honeybee viruses because they are divided into several fragments, of which only two large genome fragments have been described. Two complete CBPV complete genomes identified in this study are closely related to the previously described CBPV genome (KY937971) in Slovenia [21]. The most closely related genome from abroad is the CBPV genome from neighboring country Austria from 2018 (MK637522, MK637523). This was to be expected and is probably the result of the local spread of the viruses across the border between Slovenia and Austria. The genome of CBPV is very stable and only low heterogeneity between CBPV genomes was found, which has also been previously described as a characteristic of this extremely pathogenic bee virus [21,22,28], similar also to results observed in the collected data from the voluntary screening program for viral infections in honeybee apiaries between 2009 and 2022 in Slovenia (unpublished data).

In this study, five complete genome sequences of DWV were identified, which are the first complete genome sequences of DWV in Slovenia. Interestingly, the genomes of both genotypes A and B were found in the same sample of a clinically affected colony (DWV 341/2019), suggesting that multiple infections with genetically different strains of DWVs (very possibly also for other honeybee viruses) may also be expected, especially with Varroa mite infestations [3,16]. The results of this study also showed that different strains of the same DWV species (sample 341/2019) can be identified in positive samples, confirming a much higher complexity of the virus infection for interpretation of the positive results in the colony, which could also be reflected in a different pathology. The DWV-A genome sequences from this study are most closely related to the AY292384 genome sequence from Italy, and the DWV-B genome sequences from our study are most closely related to the sequence MN565037 from France, which are the expected results and support the previously discovered diversity of DWV in Slovenia [16].

The first complete genome sequence of LSV in Slovenia (MG918125) was determined in 2010 from a collapsed colony co-infected with CBPV [21]. In this study, we identified four new complete genome sequences of LSV in Slovenia. Comparison of the LSV strains identified in this study with different strains from GenBank confirmed the previously high diversity of LSV strains, compared to other honeybee viruses, which is probably because of the low pathogenicity of LSVs [17,22]. In Belgian honeybee colonies, multiple LSV strains were detected while screening for the new contributary factors of winter mortality of honeybees. They named it the LSV complex, where LSV3 and LSV4 were also identified like in our study and the associations were found with BQCV infection [29]. Also in apiaries in the United States, the connection with LSV2 abundance and weaker honeybee colonies was observed, respectively [30].

The first complete genome sequence of SBV in Slovenia was identified from the sample of an apparently healthy honeybee colony (LS 20/2018-ON620343), confirming the SBV infection in colony, and contamination of worker bees, probably during cleaning of the infected cells. The most closely related genome sequence is the sequence OL803870 from the Czech Republic, with which the Slovenian genome has a nucleotide identity of 98.01%, and this new strain is expanding the diversity of SBV on the phylogenetic tree (Figure 8).

The NGS method was also successfully used to identify ARV-1, BeeMLV and HPLV34 in Slovenia, which were not detected previously on our territory. These viruses were described for the first time in Slovenian honeybee colonies and reported in very few European countries. This confirms that NGS is an excellent tool for the detection of new species and strains of genetically distinct viruses in tested samples. With the increasing frequency of using NGS for detection and identification of pathogens also in honeybee samples, very interesting results (detection of co-infection of several viruses, new strains and new honeybee viruses) might be expected in the near future in this research area. ARV-1 differs strongly from other honeybee viruses due to its negative-stranded RNA genome; most other honeybee viruses have a positive-stranded RNA genome. ARV-1 has been detected in 20% of honeybee colonies, Varroa mites and bumblebees but it is not yet known whether the virus causes clinically notable disease in honeybees [19]. The first identified ARV-1 in Slovenia (the sample named 341/2019) was detected in an affected honeybee colony as a co-infection, together with three other honeybee viruses (CBPV, DWV-A, DWV-B). An additional screening study is necessary for determination of the prevalence of this virus in our territory. BeeMLV is a poorly known virus, of which only a few complete genomes from honeybee and Varroa mite samples are available in the GenBank, but its replication and pathogenicity have not yet been defined [31]. In this study, the first complete genome of BeeMLV (ON648755) from a sample of a honeybee colony in Slovenia was described because we used NGS technology for the first time in our laboratory on field samples. The BeeMLV complete genome is most closely related to the MZ821799 genome from China, which is far away, and this probably might be because of the lack of information on the prevalence of this virus from European countries. The complete genome of HPLV34 from a honeybee colony of *Apis mellifera carnica* is presented here, potentially caused by the surface contamination of honeybees. It has already been described in more than one honeybee colony of Ethiopian honeybees (*Apis mellifera simensis*) and an HPLV51 variant [32]. HPLV34 was also detected in land snails from China (KX884207) [33] and in an Asian hornet from Italy (MT747982) [34]. Additional research is needed to prove the relevance of this virus in honeybees. Detection of new honeybee strains and rapid identification of complete genomes by using NGS technology on field samples proved again the excellence of this technology also in honeybee pathology. This study mainly focused on the identification of the complete genomes of honeybee viruses in Slovenia, the genetic material, phylogeny and comparison with available sequences from GenBank, which is only small proportion of the benefits of NGS. Although NGS technology is complex and rather time consuming for analysis, several benefits and its constantly increasing number of publications in the last decade prove that NGS will be an important tool also in the honeybee virus research area in the near future. The larger set of the first twenty-two new complete genome sequences of different honeybee viruses from Slovenia is now available in GenBank, representing important new data for honeybee pathology that is interesting also for international community.

## 5. Conclusions

Twenty-two sequences of complete genomes of nine different virus species were identified and described from nine samples of infected and ten samples of healthy honeybee colonies from all over Slovenia (ABPV, n = 4, BQCV, n = 3, CBPV, n = 2, DWV, n = 5, LSV3, n = 4, SBV, n = 1, ARV-1, n = 1, BeeMLV, n = 1, HPLV34, n = 1). The first genome sequences of ABPV, BQCV, DWV and SBV in Slovenia were identified and described, and ARV-1, BeeMLV and HPLV34 were detected for the first time in honeybee samples in Slovenia. Two genetically different genome sequences of DWV (ON648742 and ON648741) were identified from the same sample of a diseased honeybee colony with 84.15% nucleotide identity and classified as genotype A and B of DWV. LSV lineage 4 was detected for the first time in Slovenia and its genome sequence was identified. The complete genome sequence of ARV-1 was detected, which is the first genome sequence of this virus species detected in honeybee samples in Slovenia. The genome sequence of BeeMLV was identified, one of five genome sequences of this virus species in the NCBI GenBank, and the genome sequence of HPLV34 is one of the first genome sequences of this species detected in honeybee samples worldwide.

## Figures and Tables

**Table 1:** The complete genome sequences of the honeybee viruses identified in this study are presented with the genome name, the name of the sample, the year, month and place of collection, the clinical status, the accession number in GenBank, the genome length and the genome coverage determined by NGS (Illumina).

Genome Name	Sample Name	Year/Month/Place of Collection	Clinical Status	Accession Number	Genome Length (bp)	Coverage
ABPV 366/2020	366	2020/Jun/Mengeš	affected	ON453877	9457	100
ABPV 377/2020	377	2020/Jul/Semič	affected	ON648739	9424	365
ABPV 376/2020	376	2020/Jun/Žalec	affected	ON648748	9452	1787
ABPV 386/2020	386	2020/Jul/Ljubljana	affected	ON648738	9440	57,446
ARV-1 341/2019	341	2019/Feb/Videm	affected	ON620344	14,585	93
BeeMLV LS13/2019	LS13	2019/Nov/Ljubljana	healthy	ON648755	6411	6055
BQCV 377/2020	377	2020/Jul/Semič	affected	ON648737	8451	35
BQCV 336/2020	336	2020/Feb/Mirna Peč	affected	ON648735	8450	4676
BQCV LS90/2019	LS90	2019/Jul/Radovljica	affected	ON648736	8450	30
CBPV RNA1 376/2020	376	2020/Jun/Žalec	affected	ON648749	3645	8964
CBPV RNA2 376/2020	376	2020/Jun/Žalec	affected	ON648750	2272	8964
CBPV RNA1 341/2019	341	2019/Feb/Videm	affected	ON648751	3617	8964
CBPV RNA2 341/2019	341	2019/Feb/Videm	affected	ON648752	2272	8964
DWV 341-2/2019	341	2019/Feb/Videm	affected	ON648742	10,145	5006
DWV LS26/2018	LS26	2018/Dec/Ljubljana	healthy	ON648744	9962	1575
DWV LS13/2018	LS13	2018/Nov/Ljubljana	healthy	ON648743	9647	7267
DWV 341-1/2019	341	2019/Feb/Videm	affected	ON648741	10,126	31,035
DWV 336/2019	336	2019/Feb/Mirna Peč	affected	ON648740	10,127	3342
LSV3 LS74/2019	LS74	2019/Mai/Ptuj	healthy	ON648746	5997	407
LSV3 LS81/2019	LS81	2019/Mai/Ljubljana	healthy	ON648747	6025	594
LSV3 LS48/2019	LS48	2019/Feb/Ljubljana	healthy	ON648745	5967	15,452
LSV4 LS21/2018	LS21	2018/Nov/M. Sobota	healthy	ON648753	5973	3375
SBV LS20/2018	LS20	2018/Nov/M.Sobota	healthy	ON620343	8787	30
HPLV34 377/2020	377	2020/Jul/Semič	affected	ON648754	1460	6055

## Data Availability

The original contributions presented in the study are included in the article and supplementary material, further inquiries can be directed to the corresponding authors.

## Supplementary Materials

The following supporting information can be downloaded at https://www.mdpi.com/article/10.3390/insects15110832/s1, Table S1: Selection of positive samples for NGS according to Cq values of ABPV, BQCV, CBPV, DWV, LSV3 and SBV and nucleotide sequences identified by Sanger sequencing method. Table S2: The list of identified 22 genome accession numbers from our study and their reference genomes [35,36,37,38] from GenBank with % nucleotide identity between each other.
