# Supplementary material for: Identification of Twenty-Two New Complete Genome Sequences of Honeybee Viruses Detected in Apis mellifera carnica Worker Bees from Slovenia
Source: Insects. 2024 Oct 24;15(11):832. doi: 10.3390/insects15110832 (PMC11594352; doi:10.3390/insects15110832)
Supplement: Supplementary file 1 [file insects-15-00832-s001.zip › insects-3219000-supplementary.pdf]

insects

MDPI

Supplementary Materials

Identification of Twenty-Two New Complete Genome Sequences of Honeybee Viruses Detected in *Apis mellifera carnica* Worker Bees from Slovenia

Laura Šimenc Kramar [1,*] and Ivan Toplak [2]

[1] Institute of Microbiology and Parasitology, Parasitology Unit, Veterinary Faculty, University of Ljubljana, Gerbičeva 60, 1115 Ljubljana, Slovenia
[2] Institute of Microbiology and Parasitology, Virology Unit, Veterinary Faculty, University of Ljubljana, Gerbičeva 60, 1115 Ljubljana, Slovenia; ivan.toplak@vf.uni-lj.si
* Correspondence: laura.simenckramar@vf.uni-lj.si

Table S1. Selection of positive samples for NGS according to Cq (cycle quantification) values of ABPV, BQCV, CBPV, DWV, LSV3 and SBV and nucleotide sequences determined by Sanger sequencing method.

No.	Virus	Sample name	Health status	Cq value	Sequence length (nt)	% identity with closest sequence (GenBank acc. No)	RNA concentration (ng/µL)
1	ABPV	324/2016	affected	10.94	433	98 % (MG737448)	507
2	ABPV	366/2020	affected	14.05	452	98 % (AF486072)	16.4
3	ABPV	386/2020	affected	4.34	434	99 % (AF486072)	17.3
4	BQCV	LS90/2019	healthy	10.95	692	100 % (MH899977)	572
5	BQCV	336/2016	affected	8.66	679	99 % (MN565034)	210
6	BQCV	377/2020	affected	14.19	690	99 % (MH899979)	408
7	CBPV	376/2020	affected	13,07	509	99 % (FJ345313)	18.4
8	CBPV	380/2020	affected	8.66	543	99 % (FJ345313)	240
9	DWV	LS13/2018	healthy	10.82	478	97 % (JF346624)	414
10	DWV	LS26/2019	healthy	8.5	431	98 % (KT004425)	521
11	DWV	341/2019	affected	9.78	479	98 % (JF346624)	614
12	LSV3	LS02/2018	healthy	15.29	482	98 % (KY465171)	591
13	LSV3	LS20/2019	healthy	13.58	578	98 % (KY465171)	513
14	LSV3	LS48/2019	healthy	12.94	504	98 % (MG918123)	283
15	LSV3	LS74/2019	healthy	13.48	578	98 % (KY465717)	68,0
16	LSV3	LS81/219	healthy	13.73	576	98 % (MG918123)	240
17	LSV3	LS84/2019	healthy	14.54	533	98 % (MG918125)	484
18	LSV3	LS21/2019	healthy	10.18	520	98 % (MG918123)	336
19	SBV3	PB/2019	affected	12.64	719	98 % (MG545286)	322

Clinically healthy bee colonies showed no clinical signs of viral infection or other signs of infectious diseases. Clinically affected colonies were colonies suspected of having a viral infection, such as dead bees in front of the hives, collapsing bees, weak colonies, trembling and/or paralysis of the bees, loss of color and hair or wing deformities.

Table S2. The list of identified 22 genome accession numbers from our study and their reference genomes form GenBank with % nucleotide identity between each other.

No.	Virus	Genome accession number	Reference genome accession number	Reference genome identity	Reference genome reference
1	ABPV	ON453877	AF150629 [37]	93 %	[35]
2	ABPV	ON648739	AF150629	93 %	[35]
3	ABPV	ON648748	AF150629	93 %	[35]
4	ABPV	ON648738	AF150629	93 %	[35]
5	ARV-1	ON620344	KY354230	100 %	No reference genome
6	BeeMLV	ON648755	KT162924	78 %	[31]
7	BQCV	ON648737	AF183905	88 %	[36]
8	BQCV	ON648735	AF183905	88 %	[36]
9	BQCV	ON648736	AF183905	88 %	[36]
10	CBPV RNA1	ON648749	EU122229	92 %	[37]
	CBPV RNA2	ON648750	EU122230	92 %	
11	CBPV RNA1	ON648751	EU122229	91 %	[37]
	CBPV RNA2	ON648752	EU122230	92 %	
12	DWV	ON648742	AJ489744	98 %	[38]
13	DWV	ON648744	AJ489744	97 %	[38]
14	DWV	ON648743	AJ489744	97 %	[38]
15	DWV	ON648741	AJ489744	84 %	[38]
16	DWV	ON648740	AJ489744	84 %	[38]
17	LSV3	ON648746	KX883223	76 %	[33]
18	LSV3	ON648747	KX883223	76 %	[33]
19	LSV3	ON648745	KX883223	76 %	[33]
20	LSV4	ON648753	KX883223	88 %	[33]
21	SBV	ON620343	AF092924	93 %	Not published
22	HPLV34	ON648754	OP972878	99 %	No reference genome

s
